# A BODIPY-Based Probe Enables Fluorogenicity via Thiol-Dependent Modulation of Fluorophore Aggregation

**DOI:** 10.3390/molecules27082455

**Published:** 2022-04-11

**Authors:** Tak Ian Chio, Akiva J. Grimaldi, Thomas I. Radford, Susan L. Bane

**Affiliations:** Department of Chemistry, Binghamton University, State University of New York, Binghamton, NY 13902, USA; tchio1@binghamton.edu (T.I.C.); agrimal2@binghamton.edu (A.J.G.); tradfor1@binghamton.edu (T.I.R.)

**Keywords:** BODIPY, maleimide, thiol, aggregation, aggregation-caused quenching, disaggregation-induced emission

## Abstract

Given the popular usage of BODIPY fluorophores in biological research, their propensity to aggregate in aqueous solution and impact their spectroscopic properties arguably warrants more attention. The probe under study herein serves as a case in point. A *para*-maleimide-substituted *meso*-phenyl BODIPY (p-MB) had previously been characterized in organic media, where its inherently high fluorescence ruled out its fluorogenic potential. Here, we have found that in aqueous solution, p-MB behaves differently, exhibiting a much-reduced fluorescence as a result of aggregation-caused quenching (ACQ). Additionally, p-MB is capable of responding to complementarily reactive substrates, including thiols and TCEP, to generate a substantial turn-on signal. The fluorescence restoration is largest when it reacts with those containing adjacent ionizable groups. By being part of a polar conjugate, p-MB assumes a disaggregated form, circumventing ACQ and unleashing up to ~1000-fold fluorescence enhancement through apparent disaggregation-induced emission (DIE). While our results support DIE as the turn-on mechanism, we found that the reactivity of the probe is much lower when it is given time to form stable aggregates. Therefore, contrary to the conventional depiction that a DIE probe works by dispersing from preformed aggregates to react with the target, our results suggest that it functions via a target-mediated *inhibition* of probe aggregation. Altogether, our work highlights the aggregation issue often faced by BODIPY-based probes and demonstrates how that can be exploited for turn-on sensing application. Furthermore, it reconstructs a different pathway for the DIE mechanism.

## 1. Introduction

Boron dipyrromethene (or 4,4-difluoro-4-bora-3a,4a-diaza-s-indacene; BODIPY) is a popular class of fluorescent dye known for its high molar absorptivity and quantum yield, small Stokes shift, and excellent photochemical stability [1]. Synthetic accessibility to substituted derivatives, modified at the *meso*, pyrrole, and/or boron positions, provides various ways to tune their spectroscopic properties [2,3]. These collective features of BODIPY invite its wide-ranging applications, where it serves as the scaffold for a plethora of biomolecular probes [4,5] and organic materials for light-emitting devices [6,7]. 

Despite its widespread usage in biology as fluorescent probes for biomolecules, BODIPY’s overall hydrophobic character confers a susceptibility to aggregate in aqueous solution. The aggregation can potentially lead to one of two fates: aggregation-induced emission (AIE) or aggregation-caused quenching (ACQ). The former enables activation of fluorescence during the aggregated state while the latter results in quenching. There have thus been deliberate efforts toward developing probes capable of AIE, while ACQ often stands as an unintended and undesired attribute [7,8].

Although it is well-accepted that BODIPY has a relatively high tendency to aggregate given its hydrophobic nature, the potential accompaniment of ACQ is arguably not sufficiently appreciated. Importantly for BODIPY, its major spectroscopic appeal, namely high absorption and emission, is lost or diminished when affected by ACQ. The effect is also most pronounced in water, the most biologically relevant medium. Consistent inclusion of water as a solvent (beyond the more typical set of organic media) to characterize BODIPY-based probes is thus important to ensure that their actual behavior in the pertinent environment is not overlooked [9].

Indeed, our encounter of the aggregation behavior of BODIPY came to our surprise from a reported probe previously deemed to have high intrinsic fluorescence. Nagano et al. synthesized three maleimide-functionalized BODIPY (MB) derivatives, in which the thiol-reactive group is attached *ortho*-, *meta*-, or *para*- to the *meso*-phenyl substituent [10]. They discovered that the *ortho*-maleimide group can act as a nearby electron acceptor to quench the donor BODIPY’s fluorescence via a photoinduced electron transfer (PET) mechanism. Reaction with thiols deactivates the PET and restores BODIPY’s fluorescence, producing a turn-on signal. 

As the *para*-derivative has the maleimide positioned furthest away from the BODIPY core, it was rationalized that it would experience the least quenching effect from PET, precluding its fluorogenic ability in response to thiols. This was supported by the *para*-derivative having the highest quantum yield among the three constitutional isomers. Notably, however, the quantum yield measurements were performed in dimethyl sulfoxide (DMSO) rather than in water where the probe would operate given its thiol labeling application. 

**Figure 1:**
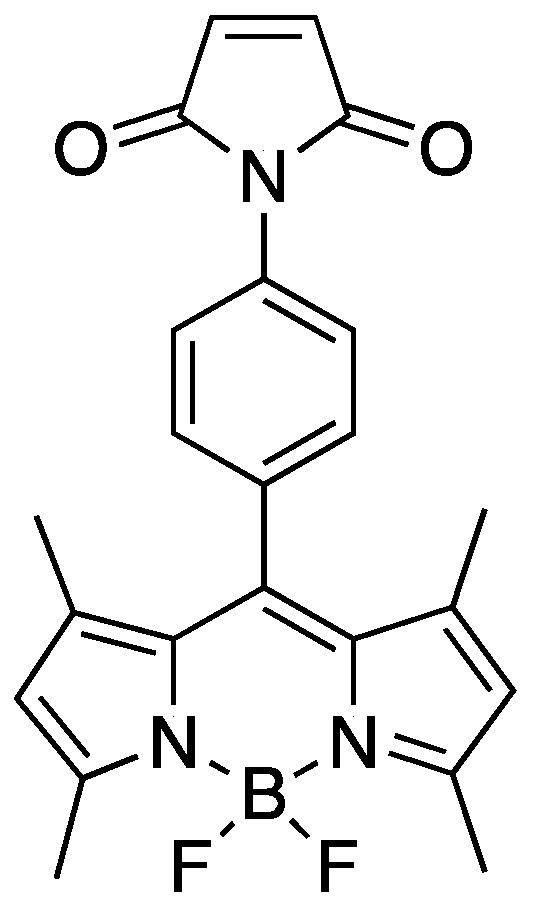
Fluorophore used in this work: *para*-maleimide BODIPY (p-MB).

In aqueous medium, we have found in this work that the *para*-maleimide BODIPY (p-MB) (Figure 1) actually demonstrates low fluorescence, which we attribute to ACQ. p-MB is furthermore capable of producing a strong turn-on signal upon reaction with thiols in a manner reminiscent of a disaggregation-induced emission (DIE) mechanism. Here, the conjugation of polar moieties discourages the probe from aggregate formation, retaining the canonically robust fluorescence of BODIPY. Altogether, this study adds appreciation to the aggregation issue commonly faced by BODIPY-based probes in aqueous conditions. It also re-instates p-MB as a fluorogenic thiol-reactive sensor, which serves as an example highlighting how the unwanted aggregation behavior of BODIPY can be exploited to enable a turn-on sensing capacity. 

## 2. Results and Discussion

### 2.1. Aggregation-Caused Quenching of p-MB in Aqueous Solution

**Figure 2:**
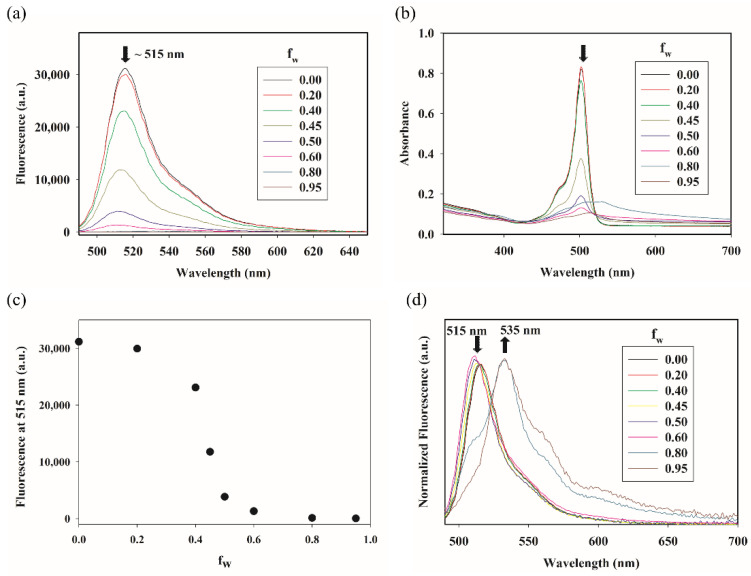
Fluorescence and absorption spectra of p-MB in DMSO–water mixture. (**a**) Fluorescence spectra of p-MB (50 μM) in DMSO solution with varying fractions of water (f_w_). (**b**) Absorption spectra of the solutions in (**a**), retaining the same color coding. (**c**) Fluorescence intensity of 50 μM p-MB at 515 nm as a function of solvent composition. (**d**) Fluorescence spectra from (**a**) were normalized to have the same intensity to illustrate the red shift in the p-MB emission maximum in mostly aqueous solution vs. DMSO. Arrows in (**a**,**b**,**d**) denote the direction of change at the specified wavelengths as f_w_ increases. The excitation wavelength for the emission spectra was 470 nm. a.u. = arbitrary unit.

The synthesis of p-MB was carried out as described previously [10]. In concordance with Nagano et al., p-MB is strongly fluorescent in neat DMSO. Additionally, it maintains a similarly strong fluorescence across various organic solvents, including acetonitrile, methanol, isopropanol, dioxane, and ethyl acetate (Supplementary Figure S1). The spectroscopic behavior of p-MB changes, however, in aqueous solvent. In particular, when we examined p-MB in different ratios of DMSO–water mixtures, there was a progressive diminution in both the absorption and the emission of p-MB (50 µM) as the volume fraction of water (f_w_) increased (Figure 2a,b). A closer look at the absorption spectra at higher f_w_ (<40% DMSO; Figure 2b) reveals a broadening of the absorption band and an elevated offset, suggesting the presence of some dye aggregates of sufficient size to scatter the incident light. The absorption maximum is also red-shifted compared to that in 100% DMSO. 

Similar to absorption, the emission weakens substantially as f_w_ increases. This holds true after taking into account the concurrent decrease in absorbance at the excitation wavelength (470 nm). Plotting the fluorescence intensity at the emission maximum in 100% DMSO (515 nm) as a function of f_w_ displays a sigmoidal relationship between the two (Figure 2c). If water was exerting a simple solvent effect on the emission intensity, the decrease would be expected to be linear [11]. To better view the spectral differences across varying f_w_, emission spectra were normalized to have the same intensity at their maxima (Figure 2d). This provides a clear illustration of the presence of at least two species in solution. The ratio of the two changes according to f_w_. One of them is strongly emissive at the expected emission maximum (515 nm) and predominates at higher % DMSO; the other exhibits a far weaker, red-shifted band at ~535 nm and predominates at higher f_w_. The bathochromic absorption and emission with increasing water content are reminiscent of the formation of J-aggregates. Our results are in line with the study by Descalzo et al., which found evidence of J-aggregates formed by 1,3,5,7-tetramethyl-8-phenyl-BODIPY in aqueous solution. This molecule shares the same structure of p-MB, except being devoid of the *para*-maleimide group [9].

**Figure 3:**
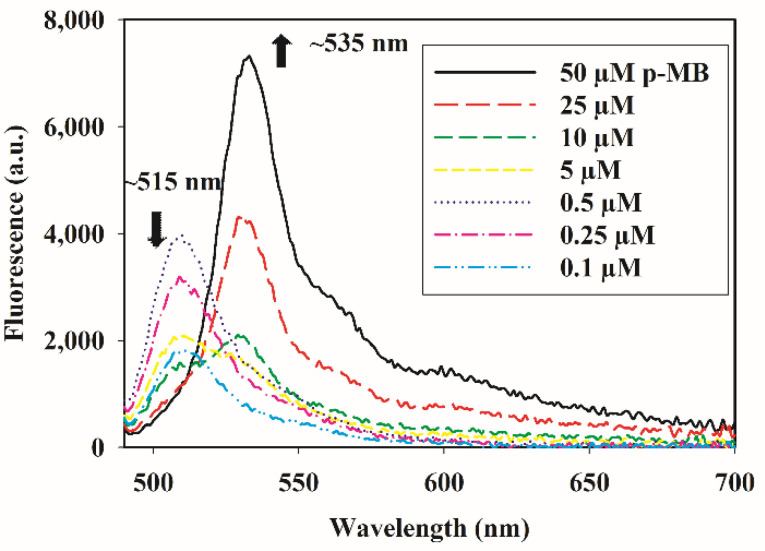
Effect of concentration on p-MB fluorescence in aqueous solution. The fluorophore stock solution in DMSO was diluted to the stated concentrations in 0.1 M PBS (0.15 M NaCl, pH 7.4). The excitation wavelength was 470 nm and the final concentration of DMSO in all solutions was 5% (*v*/*v*). Arrows denote the direction of change at the specified wavelengths as p-MB concentration increases. Note, however, that the decreasing fluorescence at 515 nm does not apply for the lower sub-micromolar concentrations. See Supplementary Material Figure S2 for spectra of sub-micromolar concentrations of p-MB (0.1–0.5 µM) without interference from the higher concentrations.

To ensure that the weakening and the bathochromic shifts of absorption and emission are not due to solvent effects but rather aggregation, we further examined the effect of dye concentration on its fluorescence in aqueous solution. Aggregation is expected to be more pronounced as the dye concentration increases. Here, the emission spectra were taken at varying concentrations of p-MB in phosphate buffered saline (PBS; pH 7.4), containing 5% DMSO. At low concentrations (0.1–0.5 µM), a single fluorescence band with a maximum at 515 nm was observed, resembling that seen in pure DMSO. As the concentration increased further, the peak at 515 nm diminished as the second peak at 535 nm arose, akin to what we had observed when f_w_ changes in the solvent (Figure 3). Therefore, at low concentration or in organic media, p-MB maintains a highly emissive monomeric state that is characterized by fluorescence at 515 nm. On the other hand, at high concentration and in aqueous solution, p-MB is subject to aggregation, undergoing ACQ with poor emission at 535 nm.

### 2.2. Inhibition of Aggregation-Caused Quenching of p-MB via Conjugation with Polar Substrates

**Figure 4:**
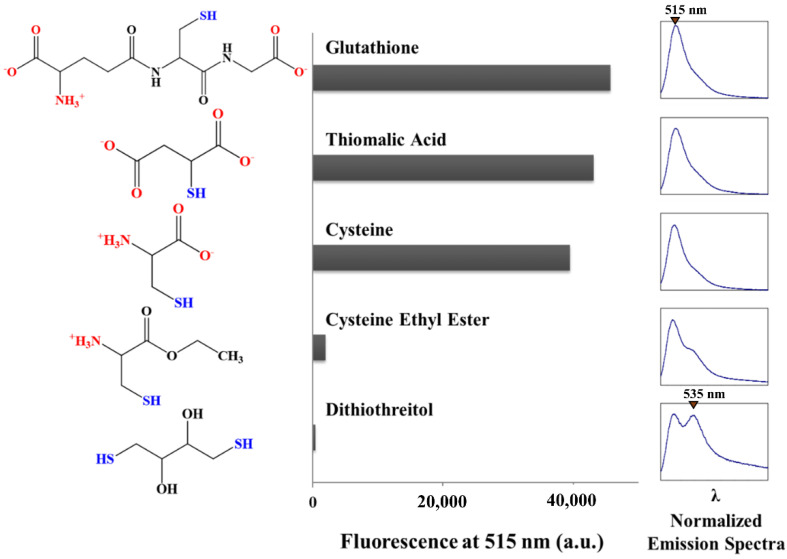
Fluorescence of p-MB after reaction with various thiols. p-MB was allowed to react with select thiol-containing molecules, the structures of which are shown in the left panel. The bar graph in the middle illustrates the fluorescence intensity of each reaction sample at 515 nm after steady state was reached. Normalized emission spectra are shown in the right panel. The emission maximum of the first peak is at ~515 nm, while that of the second peak, if present, is at ~535 nm. Samples were excited at 470 nm. The presence of the conjugates was verified by mass spectrometry (Supplementary Figure S3).

Our results thus far demonstrate that p-MB exhibits weak fluorescence in aqueous solution due to ACQ. Since ACQ is a property of the state of the fluorophore and not an intrinsic optical property of the molecule, we reasoned that the quenched fluorescence offers an opportunity to leverage, particularly for turn-on responsiveness. An increasing number of fluorogenic sensors, including BODIPY-based ones, have been developed in recent years that operate on the reversal of aggregation as a turn-on mechanism [12,13,14,15,16]. Binding or reaction with the target keeps the probe in the monomer form, leading to fluorescence recovery, or disaggregation-induced emission (DIE). Inspired by this strategy, we thus examined whether covalent reaction with thiols would restore the fluorescence of p-MB. 

p-MB was allowed to react with assorted thiol-containing compounds, including two common biological ones, namely cysteine and glutathione (GSH) (Figure 4). Under the same conditions where p-MB showed complete aggregation (50 µM in PBS with 5% DMSO), a considerable increase in fluorescence (up to ~1000-fold) was observed in the presence of GSH, thiomalic acid, and cysteine. The emission maximum also shifted back to 515 nm, which is indicative of monomer fluorescence. Meanwhile, the fluorescence increase was much less with cysteine ethyl ester and dithiothreitol (DTT). Their emission spectra showed two peaks with maxima at 515 nm and 535 nm, suggesting some persistence of aggregation. The differential signal amplification is not likely due to differences in reactivity, as GSH (pK_a_ = ~9.0), with a higher pK_a_ and presumably lower reactivity than cysteine ethyl ester (pK_a_ = ~6.5) [17], still demonstrated substantially greater fluorescence recovery. Interestingly, the ones that resulted in the greatest fluorogenic signal were those that carry two ionizable groups. Cysteine ethyl ester and DTT, on the other hand, are singly charged and uncharged, respectively. Conjugation to polar ionizable moieties therefore renders a greater propensity for the probe to disaggregate, enabling a stronger turn-on signal. 

**Figure 5:**
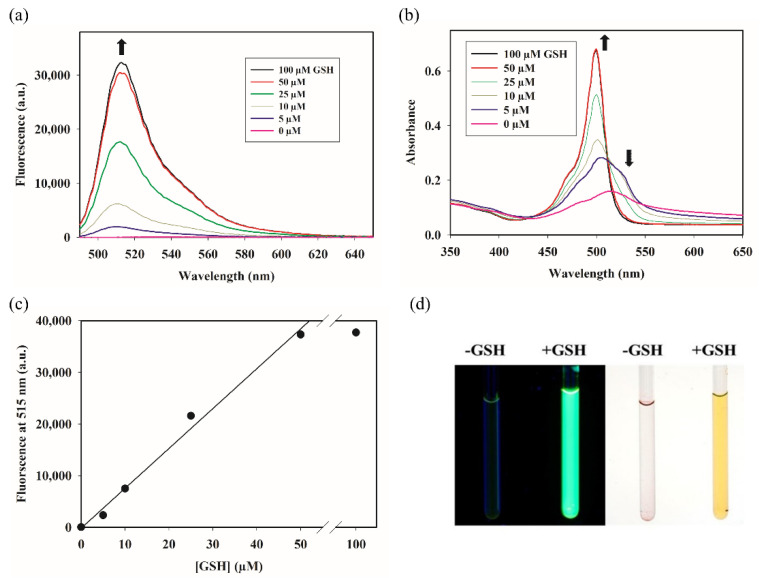
Fluorescent turn-on sensing of GSH by p-MB. (**a**) Fluorescence spectra of p-MB (50 μM) with varying concentrations of GSH in PBS containing 5% DMSO (*v*/*v*). (**b**) Absorption spectra of the solutions in (**a**), retaining the same color coding. Arrows denote the direction of change at the specified wavelengths as GSH concentration increases. (**c**) Plot of fluorescence intensity at 515 nm as a function of GSH concentration. The trendline only accounts for the concentrations up to 50 μM. The extra point at 100 μM is included to illustrate the signal saturation at an excess GSH concentration. (**d**) Image of samples containing p-MB (50 μM) in the presence or absence of GSH (500 μM) in PBS containing 5% DMSO. Left image was imaged under long wavelength UV light; right image was imaged under white light.

The generation of DIE upon conjugation with thiols demonstrates that p-MB, like its *ortho*-counterpart, can also serve as a fluorogenic probe for thiols, albeit via a different mechanism. Reaction of p-MB with increasing concentrations of GSH, for instance, exhibited a parallel increase in fluorescence and absorbance signals (Figure 5a,b). The increase in the fluorescence intensity at 515 nm was linear as a function of GSH concentration (Figure 5c). The signal plateaued when the analyte concentration exceeded that of the probe (Figure 5a–c; compare 50 µM and 100 µM GSH), indicating that the signal amplification results from a one-to-one reaction between the probe and the analyte. The limit of detection of the p-MB–GSH conjugate was found to be ~0.5 nM (Supplementary Figure S4). Imaging of the p-MB and the p-MB–GSH reaction samples under UV light illustrates the dramatic turn-on response that the probe undergoes in the presence of GSH (Figure 5d). Importantly, the thiol-dependent fluorogenicity is only applicable in aqueous solvent. A reaction of p-MB with GSH in acetonitrile did not yield a more fluorescent product (Supplementary Figure S5). This further precludes liberation of PET-mediated quenching as the fluorescence turn-on mechanism.

Finally, to ensure that the apparent DIE signal seen with the assorted substrates examined above was specific to the chemical reaction with the probe, p-MB was allowed to react with cystine or other amino acids lacking a maleimide-reactive group (Supplementary Figure S6). In all cases, their fluorescence showed negligible differences from the probe alone, with all sharing a single peak at 535 nm, which signifies complete conversion to the aggregate form. Our data thus support a target-driven disaggregation-induced response by p-MB and showcase its sensing capability.

### 2.3. p-MB as a Fluorogenic Probe for TCEP 

**Figure 6:**
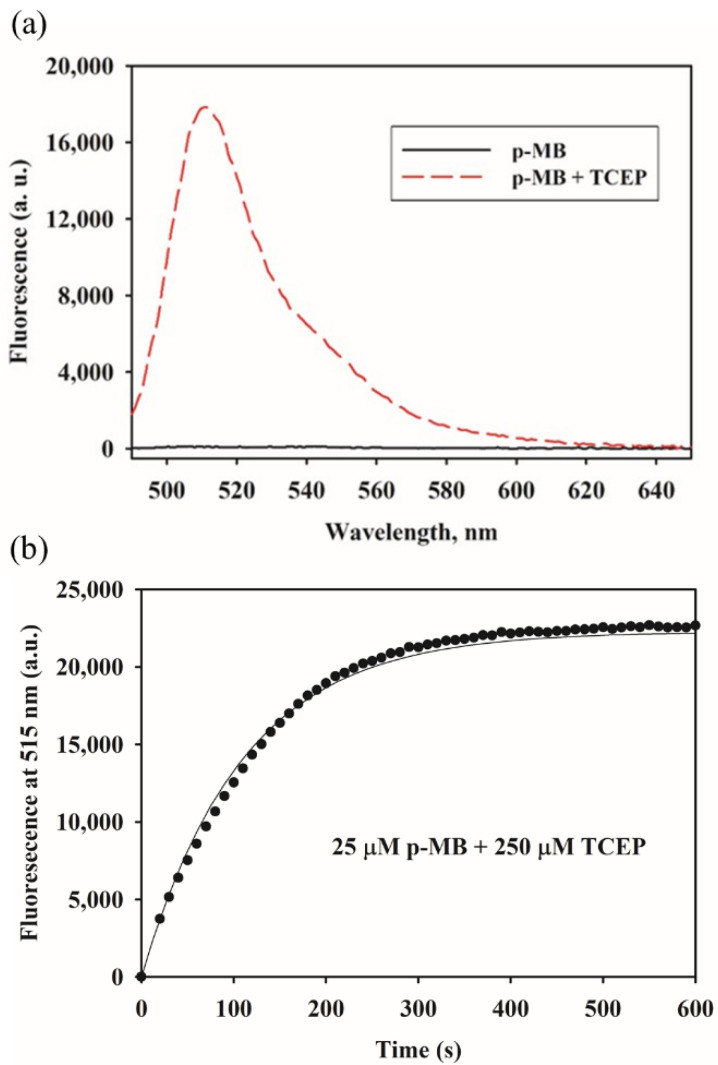
Reaction of p-MB with TCEP. (**a**) Emission spectra of p-MB (50 μM) with or without TCEP (500 μM) in PBS containing 5% DMSO (*v*/*v*). (**b**) Kinetics of reaction between p-MB (25 μM) and TCEP (250 μM). The curve was fitted to a single exponential to extract the pseudo-first order rate constant. Excitation wavelength was 470 nm. The presence of the conjugate was verified by mass spectrometry (Figure S3).

Aside from thiols, we also saw an increase in fluorescence from a non-thiol substrate, tris(2-carboxyethyl)phosphine (TCEP). TCEP is commonly used as an in situ reducing agent in reactions between thiols and maleimides, as it was formerly thought to be unreactive to the latter [18]. However, studies in recent years have demonstrated that phosphine reagents, including TCEP, do in fact react with maleimides and can compete during thiol labeling [19,20,21,22]. Further corroborating this finding, p-MB showed fluorogenic reactivity with TCEP (Figure 6a). The sizable DIE signal is likely contributed by the three ionizable carboxylate groups in TCEP that promote the disaggregated form. Monitoring the fluorescence increase over time, we were able to measure the reaction kinetics, which fits a pseudo-first order reaction mechanism and yields an estimated rate constant of ~36 M^−1^s^−1^ (Figure 6b). The slower kinetics of the maleimide–TCEP reaction relative to that with thiols (~734 M^−1^s^−1^ reported elsewhere under similar conditions [23]) is consistent with the abated interference of TCEP with maleimides compared to thiol-reducing agents [19]. 

### 2.4. Rethinking the Mechanism of DIE

**Figure 7:**
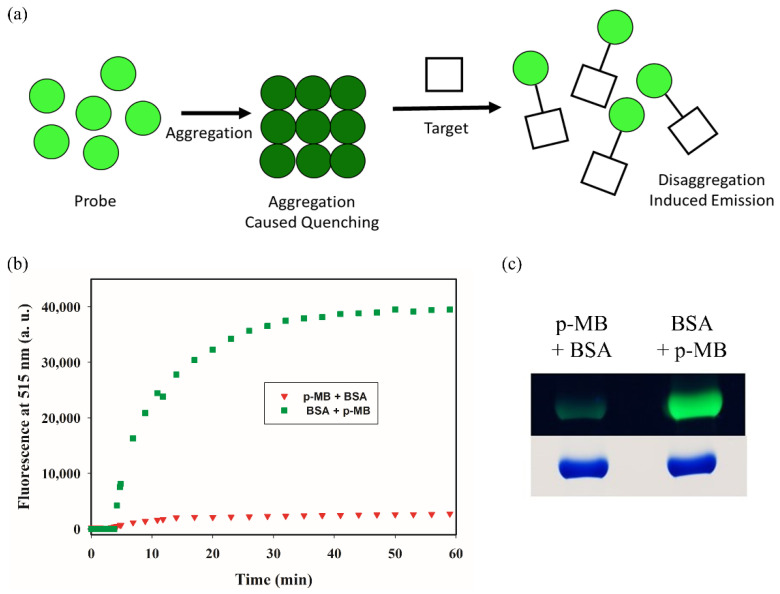
Effect of aggregation on p-MB’s reactivity. (**a**) General mechanism of DIE. (**b**) Kinetics of reaction between p-MB (50 μM) and BSA (50 μM) in PBS containing 5% DMSO (*v*/*v*). Red plot corresponds to BSA added after preincubation of p-MB in reaction buffer. Green plot corresponds to p-MB added from a stock in DMSO to the reaction buffer containing BSA. The excitation wavelength was 470 nm. (**c**) SDS-PAGE analysis of the same samples from (**b**). Top corresponds to in-gel fluorescence imaged under long-wavelength UV light, while bottom corresponds to the same gel imaged under white light after Coomassie Blue staining. Full gel images are shown in Supplementary Figure S8.

Till now, we have adopted terminologies commonly used by others to describe the turn-on mechanism of p-MB. In particular, disaggregation in DIE implies active disassembly of the probe from the aggregate as prompted by the presence of the substrate (Figure 7a). Such is the depiction often seen in schematics showcasing probes that function via the same mechanism [12,13,14,15,16]. Nevertheless, as with the way that many of these studies were carried out (based on their method descriptions [14,15,16]), our procedure thus far involved adding the probe from an organic solution into an aqueous one that already contains the substrate. In this scenario, the monomeric state of the probe during the reaction, especially in the early phase, is likely inherited from its origin from the organic medium. This is because the aggregation process, which initiates upon introduction of the probe to the aqueous milieu, is not instantaneous (vide infra). Therefore, it is unclear whether the probe, if given time to aggregate first, can readily dissociate and react with the substrate.

By monitoring the fluorescence decrease at 515 nm, we found that the aggregation of p-MB (50 µM) in aqueous solution plateaued in around 15 min (Supplementary Figure S7). To examine whether p-MB can still exert its reactivity once it assumes an aggregated state, we thus pre-incubated it in aqueous buffer for 15 min to allow aggregation to occur. Subsequently, we paired it with a protein, bovine serum albumin (BSA), as substrate. BSA contains one reactive cysteine that does not participate in disulfide bonds. Adding p-MB into a solution of BSA, as was performed previously with other small-molecule substrates, generates a substantial increase in fluorescence intensity at 515 nm (Figure 7b). On the contrary, when BSA was introduced after p-MB was given time to pre-form aggregates, the fluorescence increase was close to negligible. It should be noted that in this latter case, there is a slow yet continuous increase in fluorescence over time, suggesting that substrate-dependent disaggregation does occur, albeit very slowly. SDS-PAGE analysis of the same samples corroborated with the much-reduced extent of BSA labeling when p-MB was allowed to aggregate beforehand (Figure 7c). 

**Figure 8:**
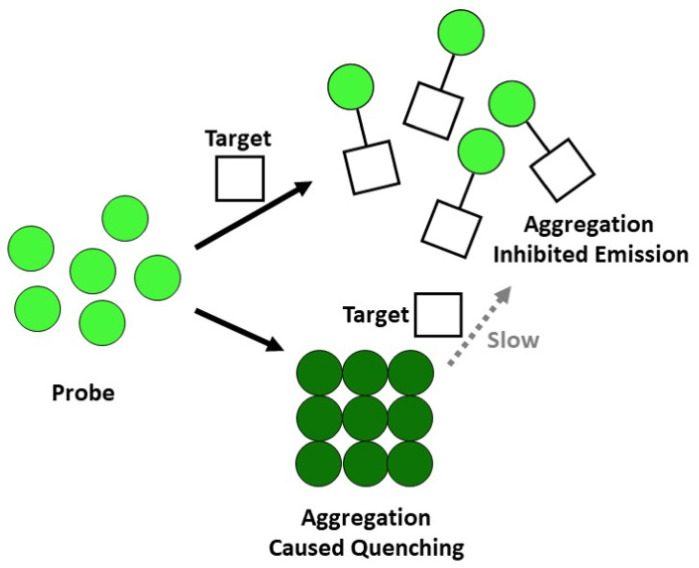
Proposed mechanism for the fluorogenic behavior of p-MB in the presence of charged thiols.

Together, our results indicate that at least for p-MB, the typical depiction of DIE, wherein the target induces disassembly of the probe from the aggregate, does not apply. Instead, a more accurate portrayal is that conjugation between the probe and the substrate inhibits aggregation (Figure 8). The extent of DIE should be unhampered if the kinetics of the conjugation reaction is faster than that of aggregation. While we cannot generalize for other DIE-based probes, our work provides an alternative perspective in thinking about how the DIE sensing mechanism works.

## 3. Conclusions

In this study, we identified and examined the aggregation behavior of p-MB, a BODIPY-based probe previously evaluated alongside its *ortho*- and *meta*-substituted counterparts as fluorescent thiol-reactive probes. Unlike the previous study, where p-MB fell short of the fluorogenic criteria due to its strong intrinsic fluorescence, we found that to be the case only in organic media. In aqueous solution and above sub-micromolar concentrations, p-MB is weakly emissive due to ACQ, with red-shifted absorption and fluorescence bands indicative of J aggregate formation. The low basal fluorescence provides room for enhancement. Reaction with thiols containing proximal ionizable groups yields up to ~1000-fold increase in fluorescence intensity. The polarity conferred on the conjugate as a result deters aggregation, enabling DIE. p-MB is thus equipped with a turn-on function that can be utilized for thiol sensing under aqueous conditions. The signal amplification is further applicable to non-thiol substrates, which we have taken advantage of to determine (to our knowledge) the first reported rate constant for the maleimide–TCEP reaction. Finally, our observation that the extent of DIE is curtailed if the probe is allowed to aggregate beforehand suggests that the probe works by target-dependent inhibition of aggregation, rather than target-induced disaggregation. Together, our work foments new thinking about the DIE turn-on mechanism. It also demonstrates the possibility of redirecting a common problem of BODIPY probes toward the benefit of fluorogenic activity. Given that the core scaffold (1,3,5,7-tetramethyl-8-phenyl-BODIPY) of p-MB was previously shown to behave similarly, it may potentially be used as a building block for developing other fluorogenic probes, the target of which may be modulated by the choice of the reactive group.

## 4. Materials and Methods

p-MB was synthesized starting with 2,4-dimethylpyrrole and 4-nitrobenzaldehyde as described by Matsumoto et al. [10], with minor modifications as noted in the synthetic protocols (Supplementary Material). The structures of the intermediate products were confirmed by ^1^H NMR spectroscopy; ^1^H, ^13^C, COSY, and HSQC NMR spectra of p-MB are included in the Supplementary Material. All other chemical reagents used were from commercial sources (Sigma Aldrich, Fisher, Acros, etc.) unless otherwise noted. Bovine serum albumin was purchased from Rockland. All NMR spectra were collected and processed on a Bruker Avance NEO 400 MHz NMR spectrometer (Billerica, MA, USA) at 298 K. All absorption and emission spectra shown here in the main manuscript were taken in a black, clear-bottom, 96-well plate on a BioTek (Winooski, VT, USA) Synergy Mx microplate reader at room temperature (RT). Equal volumes were used for all spectra. Absorbance values are relative, as they were not pathlength-corrected. The excitation wavelength for measuring fluorescence was 470 nm. Fluorescence bandpass width was 9 nm for both excitation and emission. The PMT detector sensitivity setting was adjusted depending on the experiment to ensure that the fluorescence signals fall within the dynamic range of the instrument. The PMT sensitivity setting for each set of experiment is specified in the corresponding experimental description below. 

### 4.1. Spectroscopic Properties of p-MB in Aqueous Solution

Solvent dependence: Solutions composed of different volume fractions of water and DMSO were prepared. p-MB was then added from a concentrated stock in DMSO, to a final concentration of 50 µM. After ~10 min incubation at RT, the fluorescence intensity of the samples at 515 nm was read using a microplate reader. The PMT sensitivity setting was 60%.

Concentration dependence: A p-MB stock in DMSO was serially diluted to generate stocks of varying concentrations. The same volume of each was added to PBS (0.1 M sodium phosphate, pH 7.4), to a final percent DMSO of 5% (*v*/*v*). Final concentrations of p-MB ranged from 0.1 to 50 µM. Absorption and emission spectra were taken ~20 min after addition into PBS. The PMT sensitivity setting was 100%.

### 4.2. Reaction of p-MB with Thiol Substrates in Aqueous Solution

Reaction with thiol substrates: p-MB was added from a concentrated stock in DMSO to a solution of substrates or non-substrates in PBS. Final concentrations of p-MB and the pairing substrate/non-substrate were 50 µM and 500 µM, respectively; final percent DMSO was 5% (*v*/*v*). Absorption and emission spectra were taken after the fluorescence intensity at 515 nm had plateaued, indicating completion of reaction. The PMT sensitivity setting was 60%. The fluorescence fold increase was determined by taking the ratio of the fluorescence intensity of the p-MB–thiol conjugate to that of p-MB at 515 nm.

Kinetics of p-MB with TCEP: p-MB was added from a concentrated stock in DMSO to a solution of TCEP in PBS. Final concentrations of p-MB and TCEP were 25 µM and 250 µM, respectively; final percent DMSO was 5% (*v*/*v*). Kinetics was monitored by measuring the fluorescence intensity at 515 nm over time (excitation wavelength: 470 nm). The PMT sensitivity setting was 60%.

Reaction with different concentrations of GSH: p-MB was added from a concentrated stock in DMSO to a solution of varying concentrations of GSH in PBS. Final concentration of p-MB was 50 µM and that of GSH ranged from 0 to 100 µM; final percent DMSO was 5% (*v*/*v*). Samples were incubated at RT for 30 min before measuring their fluorescence at 515 nm. The PMT sensitivity setting was 60%.

### 4.3. Comparison of Reaction Kinetics with or without Preformed Aggregates of p-MB

The reaction of p-MB with BSA (50 µM each) was carried out in two different ways in parallel. In one sample, p-MB was added from a concentrated stock in DMSO to a solution with BSA in PBS. In another sample, BSA was added to a solution of p-MB, which was pre-incubated ~20 min beforehand to allow aggregation to take place. Kinetics of the two samples were monitored by measuring the fluorescence intensity at 515 nm over time (excitation wavelength: 470 nm). The PMT sensitivity setting was 60%. The same samples were subsequently analyzed by SDS-PAGE. 

## Data Availability

Not applicable.

## Supplementary Materials

The following supporting information can be downloaded at: https://www.mdpi.com/article/10.3390/molecules27082455/s1, Supplementary Figures S1–S8, synthetic procedure for preparing p-MB, NMR spectra of p-MB. Figure S1: Absorbance and fluorescence emission spectra of p-MB in various solvents. Figure S2: Effect of concentration on p-MB fluorescence in aqueous solution. Figure S3: LC-MS chromatograms and mass spectra for pure p-MB and products of p-MB reaction with thiols and TCEP. Figure S4: LOD of p-MB–GSH. Figure S5: Spectroscopic comparison of p-MB and p-MB–GSH conjugate in acetonitrile. Figure S6: Fluorescence of p-MB in the presence of select compounds lacking a maleimide reactive group. Figure S7: Fluorescence of p-MB in aqueous buffer over time. Figure S8: Original SDS-PAGE gels for Figure 7c.

## Sample Availability

Samples of p-MB are available from the authors.
