# Supplementary material for: A BODIPY-Based Probe Enables Fluorogenicity via Thiol-Dependent Modulation of Fluorophore Aggregation
Source: Molecules. 2022 Apr 11;27(8):2455. doi: 10.3390/molecules27082455 (PMC9031299; doi:10.3390/molecules27082455)

Supplementary Material

A BODIPY-based probe enables fluorogenicity via thiol-dependent modulation of fluorophore aggregation

Tak Ian Chio, Akiva J. Grimaldi, Thomas I. Radford and Susan L. Bane

Table of Contents

Figure S1. Absorbance and fluorescence emission spectra of p-MB in various solvents 2

Figure S2. Effect of concentration on p-MB fluorescence in aqueous solution 3

Figure S3. LC-MS chromatograms and mass spectra for pure p-MB and products of p-MB reaction with thiols and TCEP 6

Figure S4. LOD of p-MB–GSH 7

Figure S5. Spectroscopic comparison of p-MB and p-MB–GSH conjugate in acetonitrile 8

Figure S6. Fluorescence of p-MB in the presence of select compounds lacking a maleimide reactive group 9

Figure S7. Fluorescence of p-MB in aqueous buffer over time 9

Figure S8. Original SDS-PAGE gels for Figure 7c 10

Synthesis of p-MB 11

NMR Spectra of p-MB 13

(a)

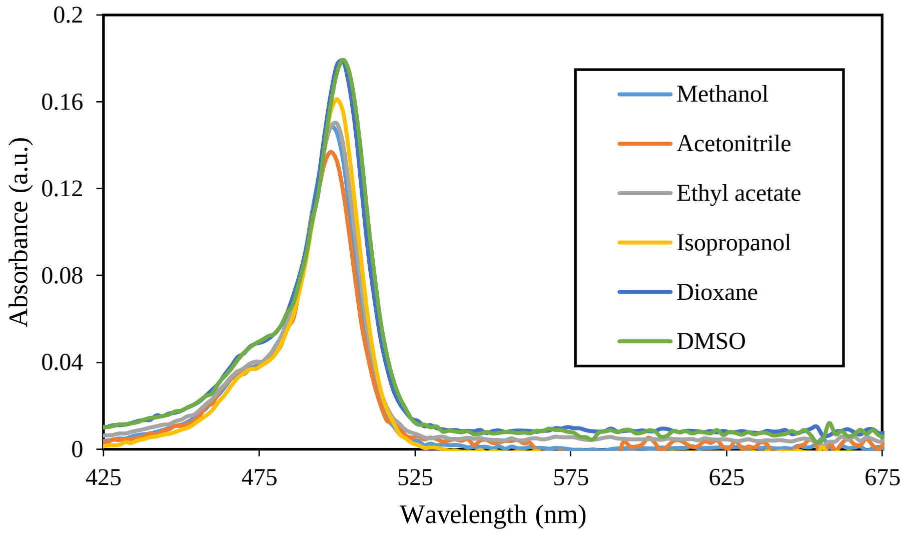

(b)

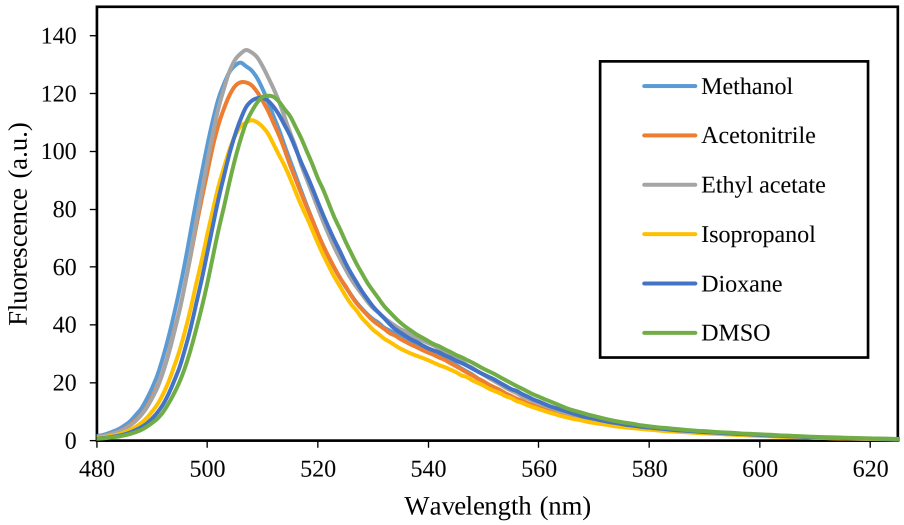

Figure S1. Absorbance and fluorescence emission spectra of p-MB in various solvents

A stock solution of p-MB in methanol was prepared. For each sample, 10 μL aliquots of the stock were evaporated, then dissolved in 1 mL of methanol, acetonitrile, ethyl acetate, isopropanol, dioxane, or DMSO. Absorption spectra were collected using an HP 8452A spectrophotometer equipped with Olis software. Emission spectra were collected using a Horiba Jobin Yvon FluoroMax-3 Spectrofluorometer using a 2x10 mm quartz cuvette, oriented such that the excitation light passed through the short path to avoid an inner filter effect. The excitation wavelength was 470 nm, and the excitation and emission slit widths were 3 nm and 2 nm, respectively. Background of the appropriate solvent was subtracted from each spectrum. (a) Absorbance spectra. (b) Fluorescence emission spectra. The spectra displayed have been normalized to equal optical density at the excitation wavelength, so the emission intensities are reflective of the relative quantum yields in each solvent. a.u. = arbitrary unit.

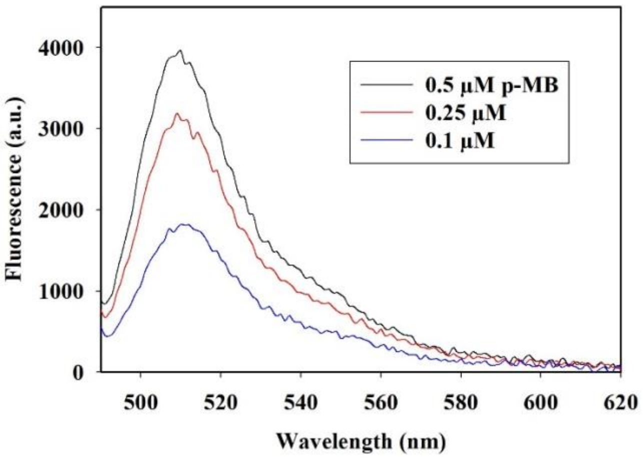

Figure S2. Effect of concentration on p-MB fluorescence in aqueous solution

The fluorophore stock solution in DMSO was diluted to the stated concentrations in 0.1 M PBS (0.15 M NaCl, pH 7.4). Emission spectra were taken in the BioTek Synergy Mx microplate reader, exciting at 470 nm. The PMT sensitivity setting was 100%. a.u. = arbitrary unit.

LC-MS confirmation of p-MB conjugates

Solutions containing p-MB (50 µM) and one of each of the thiols: glutathione (GSH), cysteine, cysteine ethyl ester, and dithiothreitol (DTT) (all 50 µM) were prepared in 10 mM PBS (pH 7.4) containing 5% DMSO. Another solution containing 50 µM p-MB and 100 µM TCEP was prepared under the same conditions. Solutions were allowed to react, then filtered through a 0.2-micron filter. The filters were washed with acetonitrile, and these portions were analyzed by LC-MS. The same procedure was carried out with a 50 µM solution of pure p-MB.

LC-MS analysis was performed a Shimadzu LCMS 2020 single quadrupole instrument. A COMOSIL C18-MS-II 2.0 × 50 mm, 2.5 µM column (Nacalai USA) was used. The flow rate was 0.2 mL/min, and the column temperature was 55 °C. The mobile phases were water with 0.1% formic acid (solvent A) and acetonitrile with 0.1% formic acid (solvent B). Each run used 100% A from 0 to 1 min, followed by a gradient to 100% B from 1 to 12 min, a hold at 100% B from 12 to 15 min, a gradient back to 100% A from 15 to 18 min, and finally a column wash with 100% A from 18 to 23 min. The absorbance detector was set at 498 nm for the LC data. Chromatograms and mass spectra were generated with Shimadzu LabSolutions software.

(a) LC chromatogram of p-MB

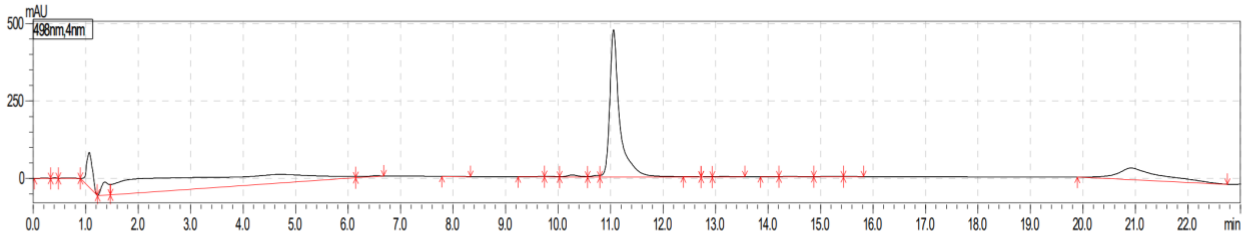

(b) Mass spectrum of p-MB for LC peak at retention time (RT) of ~11 min (positive ion mode); expected: 420 m/z $[M+H]^+$

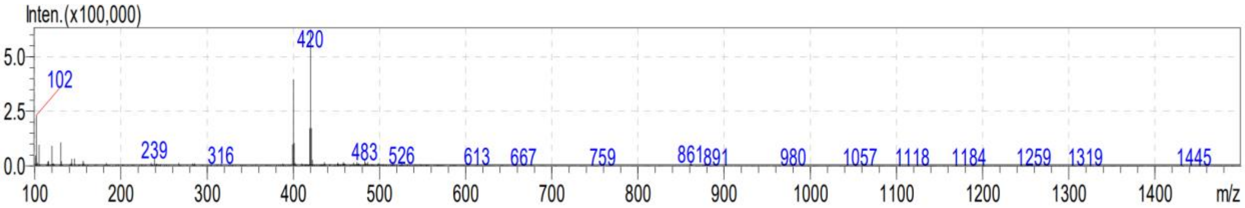

(c) LC chromatogram of p-MB–GSH reaction

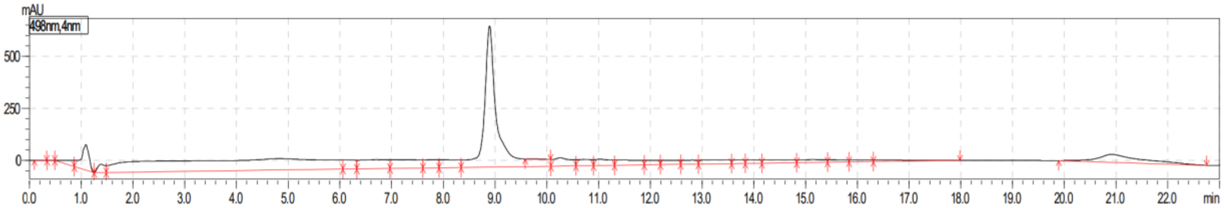

(d) Mass spectrum of p-MB−GSH reaction for LC peak at RT ~9 min (negative ion mode); expected: 725 m/z [M-H]⁻

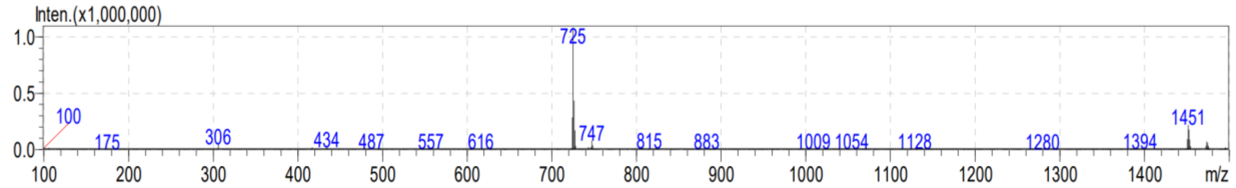

(e) LC chromatogram of p-MB−cysteine reaction

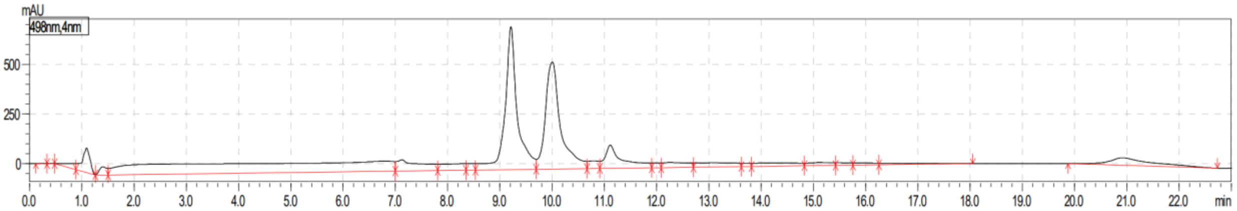

(f) Mass spectrum of p-MB−cysteine reaction for LC peak at RT ~9 min (positive ion mode); expected: 541 m/z [M+H]⁺

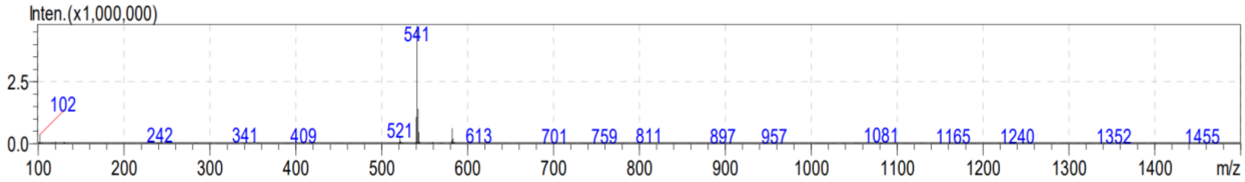

(g) LC chromatogram of p-MB−cysteine ethyl ester reaction

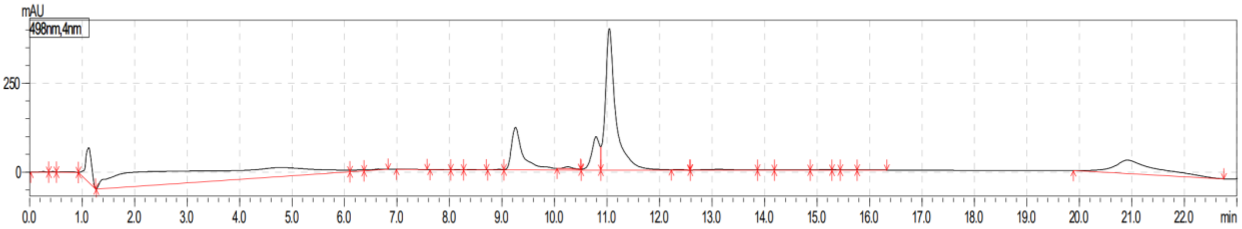

(h) Mass spectrum of p-MB−cysteine ethyl ester reaction for LC peak at RT ~9 min (positive ion mode); expected: 569 m/z [M+H]⁺

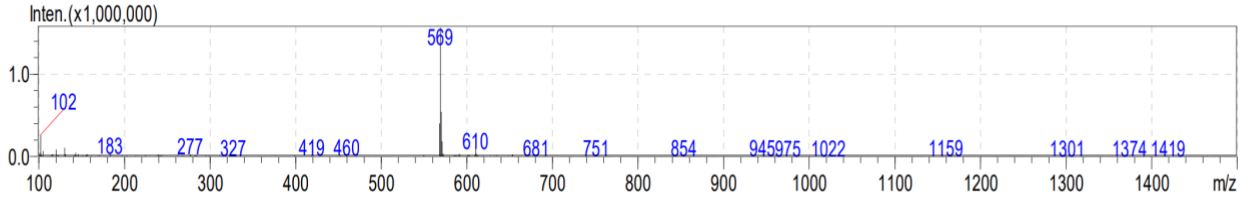

(i) LC chromatogram of p-MB–DTT reaction

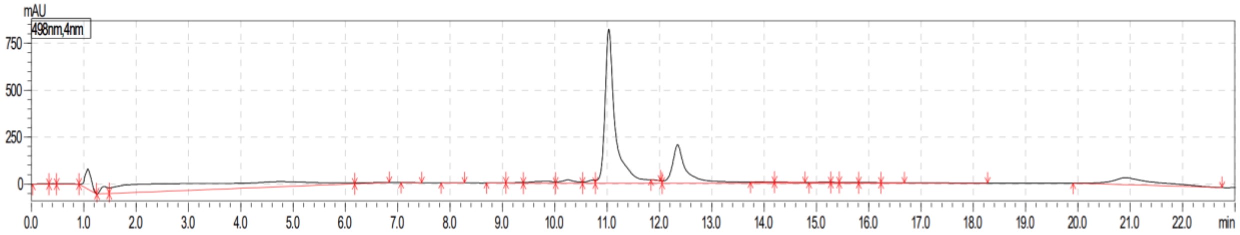

(j) Mass spectrum of p-MB–DTT reaction for LC peak at RT ~12 min (negative ion mode); expected: 572 m/z [M-H]⁻

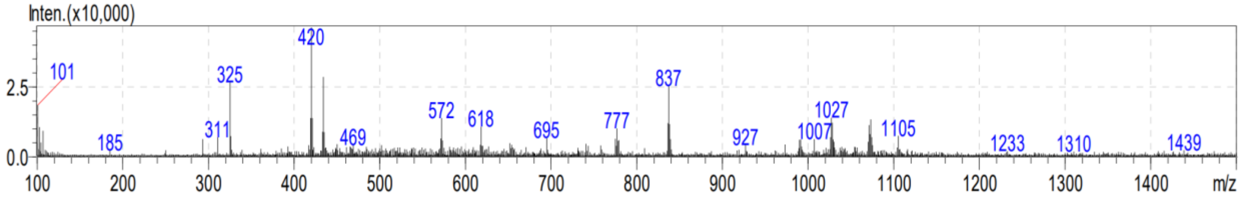

(k) LC chromatogram of p-MB–TCEP reaction

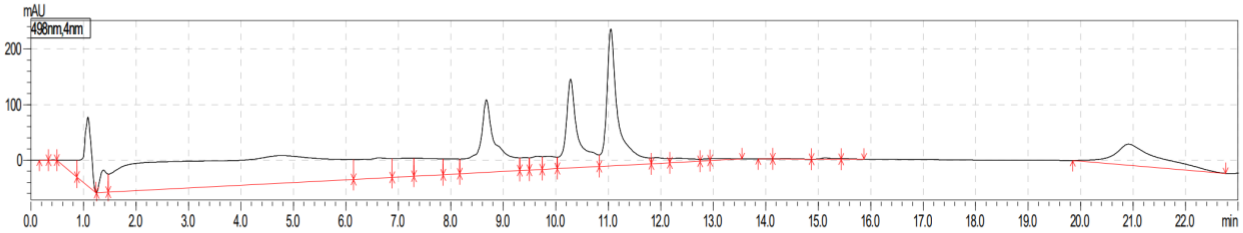

(l) Mass spectrum of p-MB–TCEP reaction for LC peak at RT ~8.5 min (positive ion mode); expected: 670 m/z [M+H]⁺

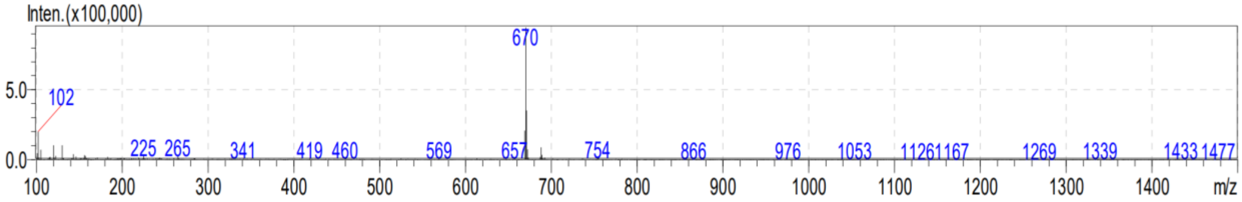

Figure S3. LC-MS chromatograms and mass spectra for pure p-MB and products of p-MB reaction with thiols and TCEP

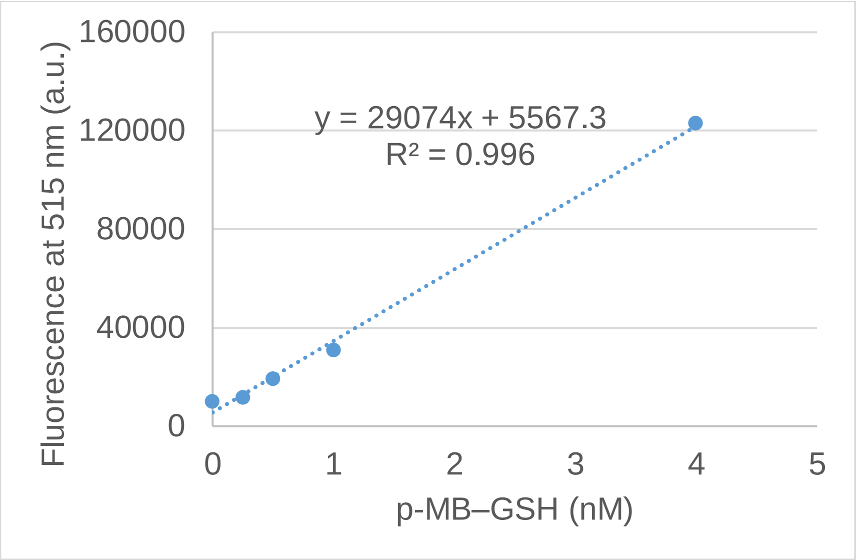

Figure S4. LOD of p-MB–GSH

A solution containing 50 μM p-MB and 75 μM GSH was prepared in 10 mM PBS (pH 7.4) containing 5% DMSO, yielding a solution containing 50 μM of the p-MB GSH conjugate. The p-MB–GSH was then diluted into varying concentrations. Emission spectra were collected using the FluoroMax-3 spectrofluorometer using a 2x10 mm quartz cuvette, oriented such that the excitation light passed through the short path to avoid an inner filter effect. The excitation wavelength was 470 nm, and the excitation and emission slit widths were 5 nm. The limit of detection (LOD) was calculated as described by Shrivastava et al.[1] using Microsoft Excel. The LOD was determined to be 0.5 nM.

[1] Shrivastava, A., Gupta, V.B., "Methods for the determination of limit of detection and limit of quantitation of the analytical methods." *Chron Young Sci* 2011, 2, 21-5

(a)

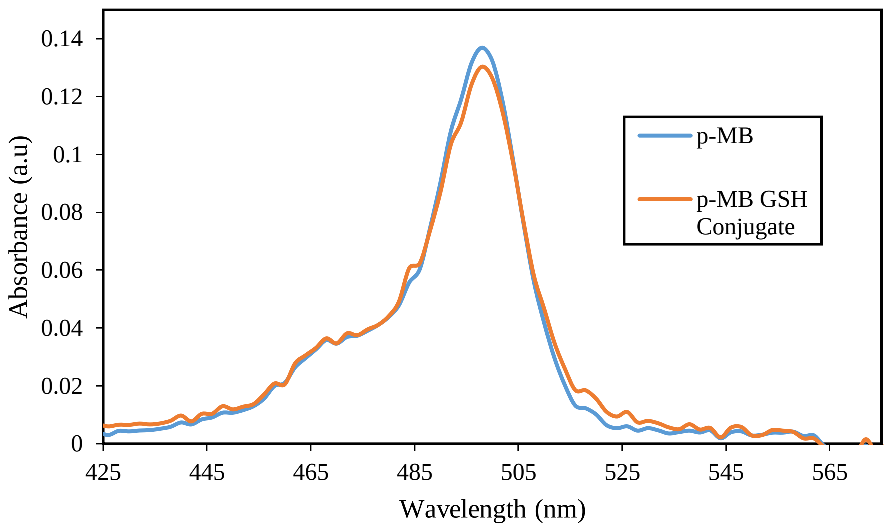

(b)

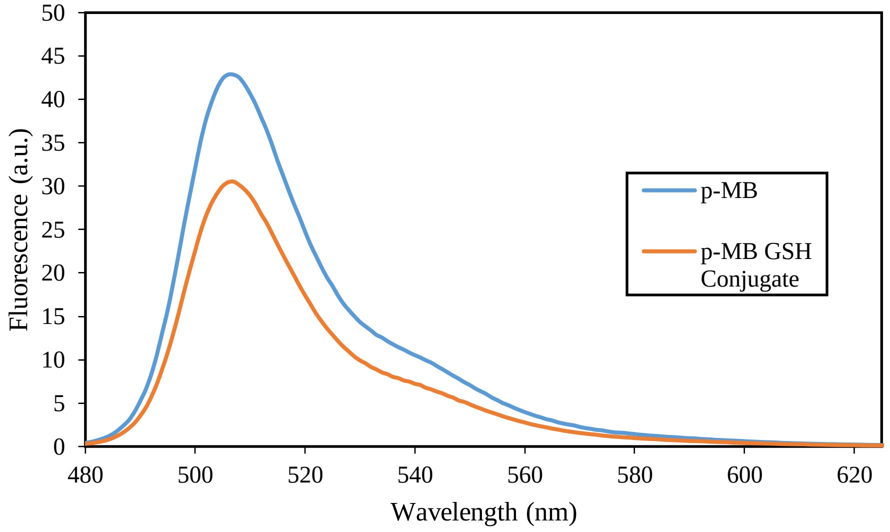

Figure S5. Spectroscopic comparison of p-MB and p-MB−GSH conjugate in acetonitrile

Solutions containing pure p-MB or pure p-MB−GSH conjugate (identity and purity confirmed by LC-MS) were prepared in acetonitrile and diluted to concentrations that yielded the same absorbance at 470 nm. (a) Absorption spectra of p-MB and its conjugate with GSH. (b) Emission spectra of the solutions in (a) were collected using a 2x10 mm quartz cuvette, oriented such that the excitation light passed through the short path to avoid an inner filter effect, in the FluoroMax-3 spectrofluorometer. The excitation wavelength was 470 nm, and excitation and emission slit widths were 3 nm and 2 nm, respectively. a.u. = arbitrary unit.

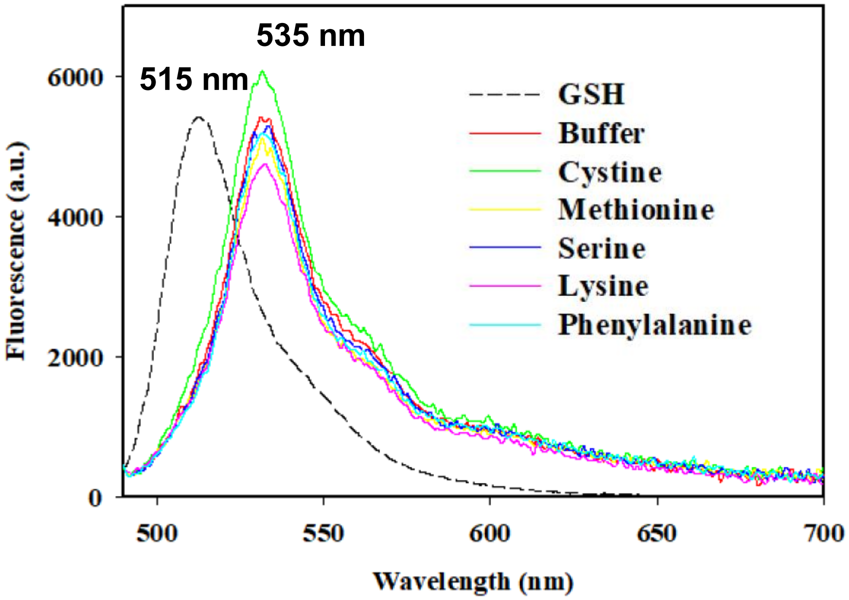

Figure S6. Fluorescence of p-MB in the presence of select compounds lacking a maleimide reactive group

The fluorescence spectrum of p-MB post-reaction with GSH (dash plot) was normalized to similar intensity to all remaining spectra to illustrate the difference in emission maxima when allowed to react with reactive (515 nm) vs. non-reactive (535 nm) substrates. Emission spectra were taken using the BioTek microplate reader, exciting at 470 nm. The PMT sensitivity setting was 100%. a.u. = arbitrary unit.

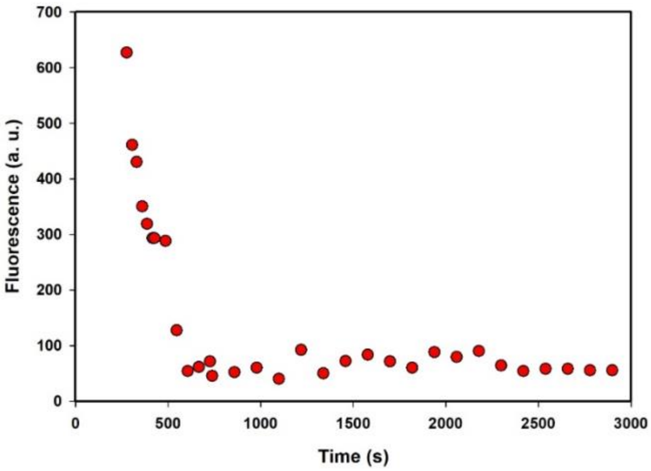

Figure S7. Fluorescence of p-MB in aqueous buffer over time

p-MB (50 μM final concentration) was added into 10 mM sodium phosphate buffer containing 1 mM EDTA and 5% DMSO. Fluorescence at 515 nm was measured over time. Data were collected using the BioTek microplate reader, exciting at 470 nm. The PMT sensitivity setting was 60%. a.u. = arbitrary unit.

(a)

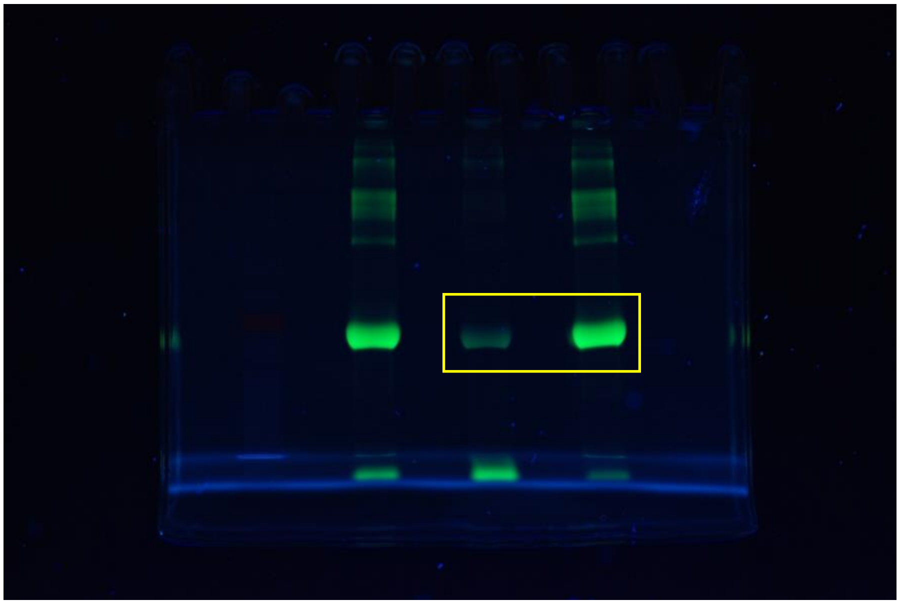

(b)

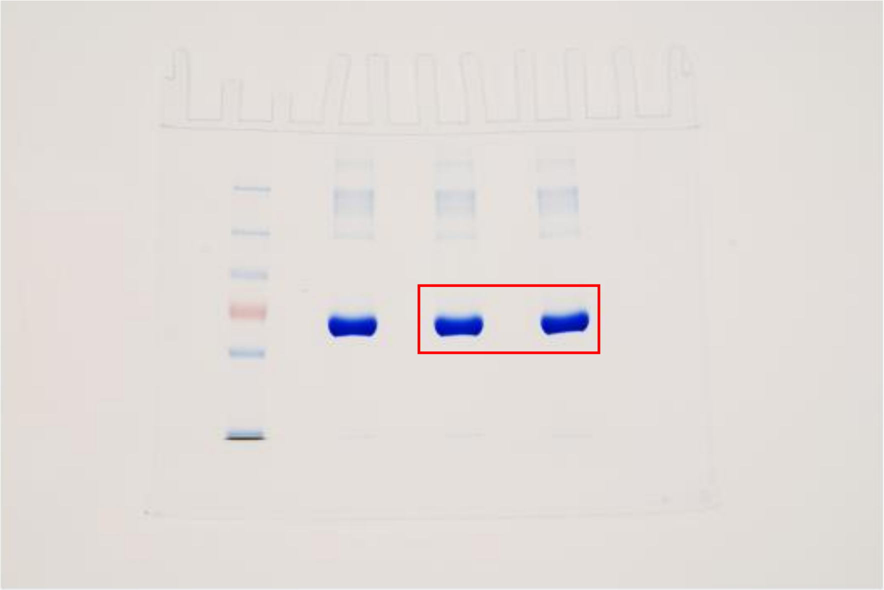

Figure S8. Original SDS-PAGE gels for Figure 7c

The SDS-PAGE gels used for Figure 7(c) are shown here in their entirety. (a) The gel was first photographed using a long wavelength UV lamp for irradiation. The lanes used in the illustration in the manuscript are shown in the yellow box. (The other lane is from a different experiment). Excess fluorophore can be seen with the dye front. (b) The same gel was then stained with Coomassie blue, destained and photographed under white light. The lanes used in the illustration in the manuscript are shown in the red box.

Synthesis of p-MB

p-MB was prepared according to the following scheme, based on reference 10.

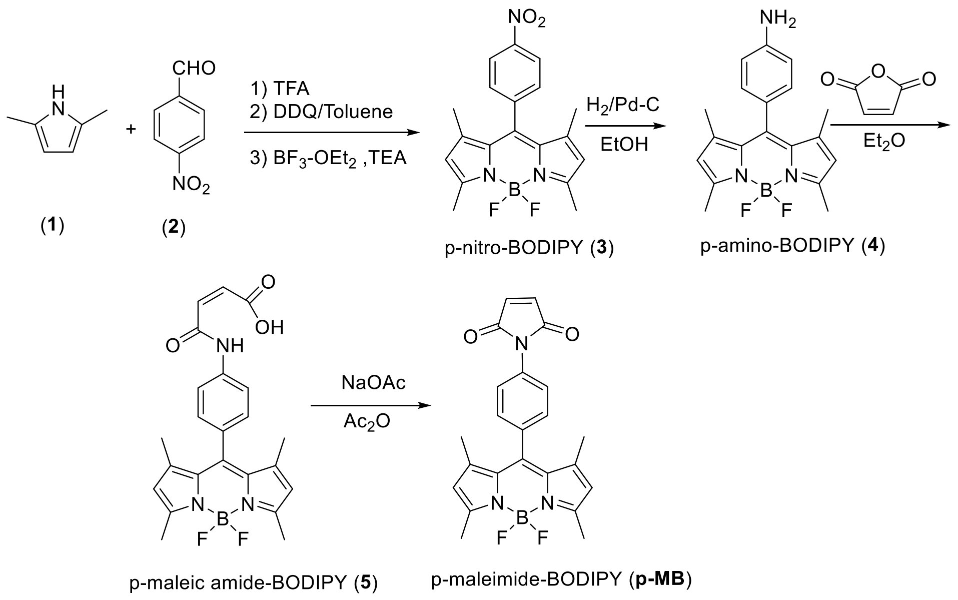

p-Nitro BODIPY (3). 2,4-Dimethylpyrrole (**1**, 2.16 mL, 21.1 mmol) and 4-nitrobenzaldehyde (**2**, 1.87 g, 12.4 mmol) were added to dichloromethane (800 mL) in a 1L round-bottom flask. The mixture was bubbled with nitrogen, and trifluoroacetic acid (0.19 mL, 2.47 mmol) was added. The reaction was stirred for 1.5 hours. The resulting solution was washed with 0.1 M NaOH (200 mL) and then water (200 mL), dried over anhydrous sodium sulfate and filtered, and the solvent was evaporated on a rotary evaporator. The resulting product was used immediately. The product was redissolved in toluene (50 mL), and DDQ (2.79 g, 11.1 mmol) was added to the mixture. After the mixture stirred for 10 minutes, triethylamine (8 mL) and boron trifluoride etherate (7 mL) were added. The mixture was stirred for 2 hours, poured into water, and extracted with toluene. The toluene solution was extracted three times with 100 mL portions of water, and the solvent was evaporated on a rotary evaporator. The residue was redissolved in chloroform and applied to a silica gel chromatography. Note the crude product can be quite insoluble / saturate the chloroform, depending on the size of the column multiple columns may be necessary. Elution with 70% chloroform in hexane (v/v) yielded **3** (664 mg, 14.4%). 1H NMR (600 MHz, CDCl3): δ 8.38 (d, 2H, J = 8.65 Hz), 7.53 (d, 2H, J = 8.58 Hz), 6.01 (s, 2H), 2.56 (s, 6H), 1.36 (s, 6H).

p-Amino BODIPY (4). Compound **3** (156 mg, 0.42 mmol) was dissolved in 25 ml of ethanol and dichloromethane (1:1 by volume) in a round bottom flask equipped with a stir bar. The solution was bubbled with nitrogen for 10 minutes followed by the addition of palladium on carbon (17 mg, 10% mol). The solution was degassed three times and the atmosphere replaced with hydrogen gas. The mixture was stirred for 12 hours. The solution was filtered through celite and washed with dichloromethane. The solvent was reduced under pressure to obtain the product 95% pure by NMR (135.3 mg, 87% yield). 1H NMR (600 MHz, CDCl3): δ 6.99 (d, 2H, J = 8.28 Hz), 6.76 (d, 2H, J = 8.29 Hz), 5.96 (s, 2H), 3.83 (s, 2H), 2.54 (s, 6H), 1.49 (s, 6H).

p-Maleic amide BODIPY (5). Maleic anhydride (23.5 mg, 0.24 mmol) was dissolved in 270 µl of diethyl ether in a round bottom flask equipped with a stir bar. The flask was fitted with an addition funnel containing a solution of compound **4** (80.4 mg, 0.24 mmol) in 5 ml of chloroform. The aniline solution was added dropwise over a period of 30 minutes while stirring. The mixture was then stirred for an additional hour. The resulting suspension was cooled to 15 ºC and vacuum filtered for the maleic acid product (62.6 mg, 60.3% yield). 1H NMR (600 MHz, CDCl3): δ 10.56 (s, 1H), 8.31 (s, 1H), 7.82 (d, 2H, J = 8.37 Hz), 7.32 (d, 2H, J = 8.27 Hz), 6.50 (d, 1H, J = 12.08 Hz), 6.34 (d, 1H, J = 12.00 Hz), 6.18 (s, 2H), 2.45 (s, 6H), 1.40 (s, 6H).

p-Maleimide BODIPY (p-MB). Sodium acetate (10 mg, 0.1 mmol) was added to 600 µl of acetic anhydride in a round bottom flask equipped with a stir bar. Compound **5** (50.0 mg, 0.1 mmol) was then added to the solution. The suspension was stirred and heated to 100 ºC for 30 minutes. The mixture was allowed to cool to room temperature then was crashed into 600 µl of ice-cold water. The resulting precipitate was vacuum filtered and washed with 100 µl of ice-cold water x3 and 100 µl of n-hexanes x1 to obtain the product (35.6 mg, 74.3% yield). 1H NMR (600 MHz, CDCl3): δ 7.57 (d, 2H, J = 8.29 Hz), 7.40 (d, 2H, J = 8.29 Hz), 6.89 (s, 2H), 5.99 (s, 2H), 2.56 (s, 6H), 1.43 (s, 6H).

NMR Spectra of p-MB

All NMR spectra were collected on a Bruker Avance NEO 400 spectrometer. 1H, 13C, COSY, and HSQC spectra were collected for a saturated solution of p-MB in DMSO-d6. The spectra were referenced to the 1H and 13C signals for DMSO-d6 of 2.50 ppm and 39.52 ppm, respectively.

1H NMR spectrum of p-MB

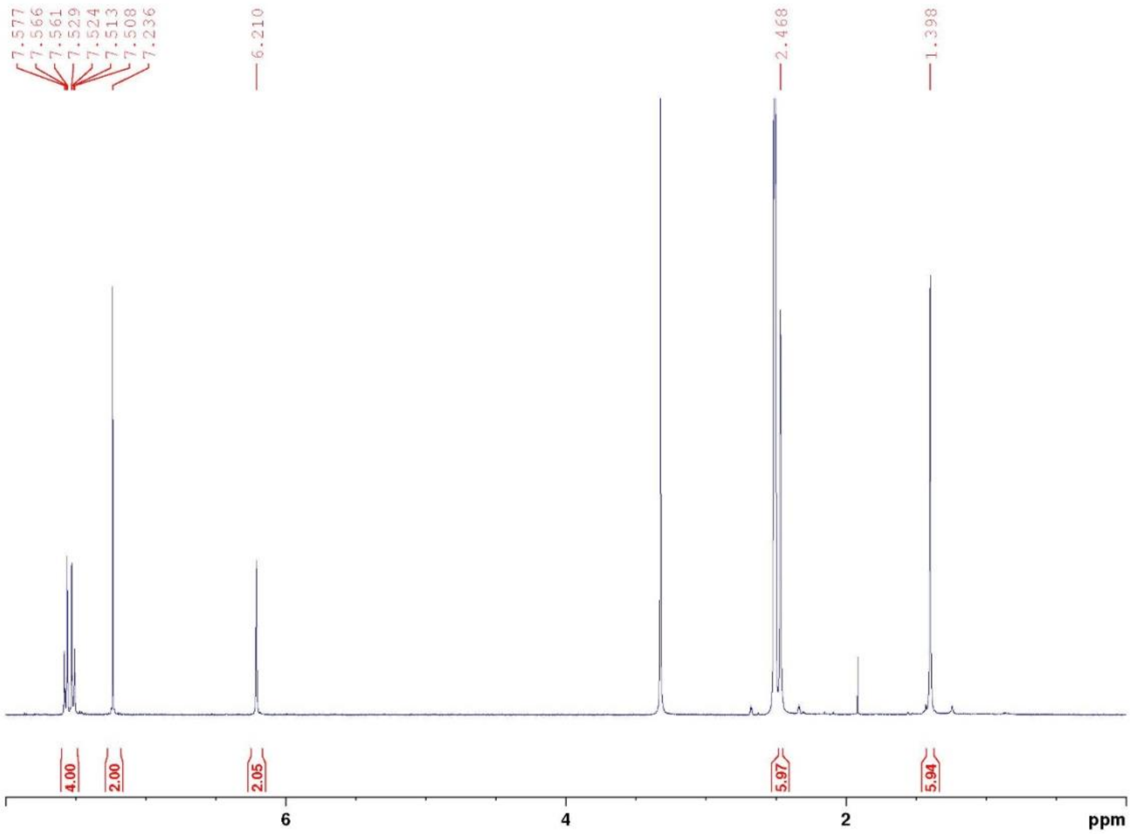

13C NMR spectrum of p-MB

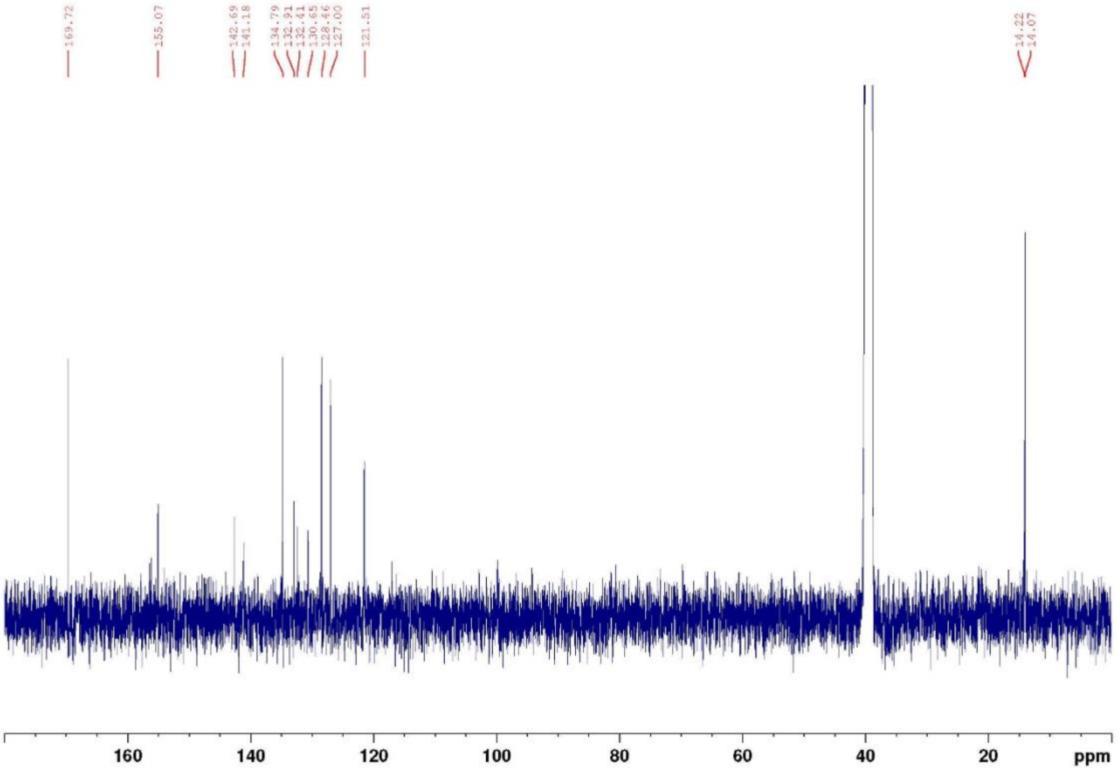

COSY NMR spectrum of p-MB

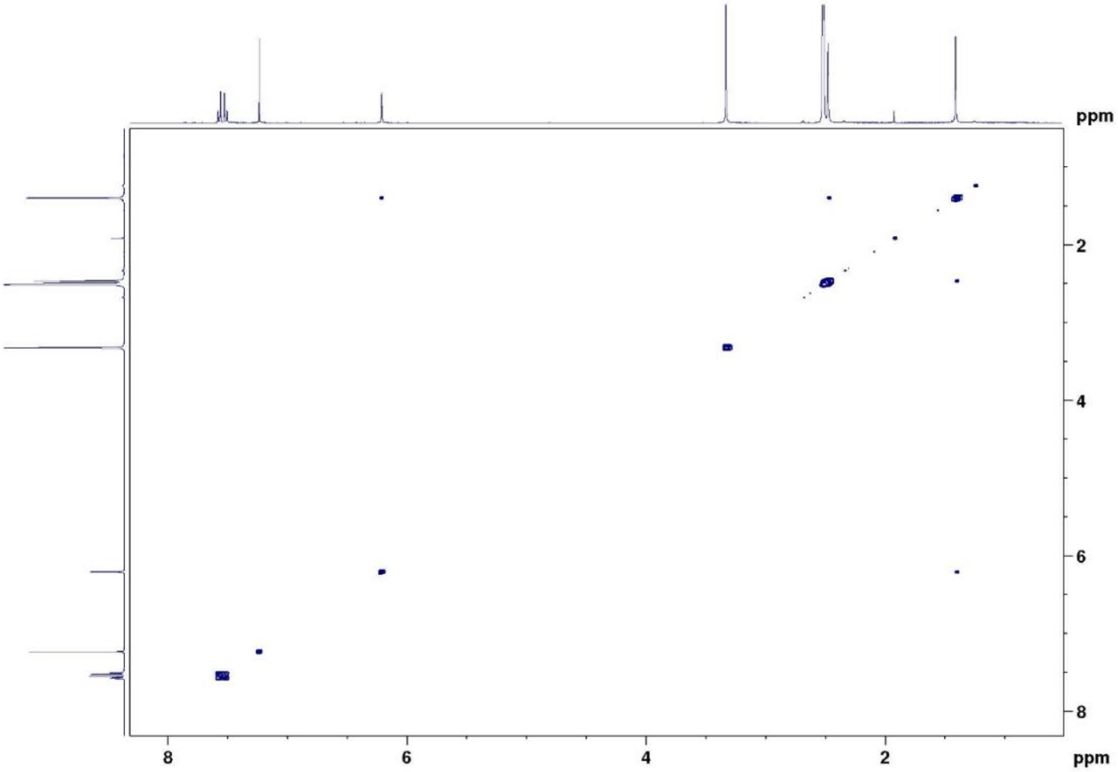

HSQC NMR spectrum of p-MB

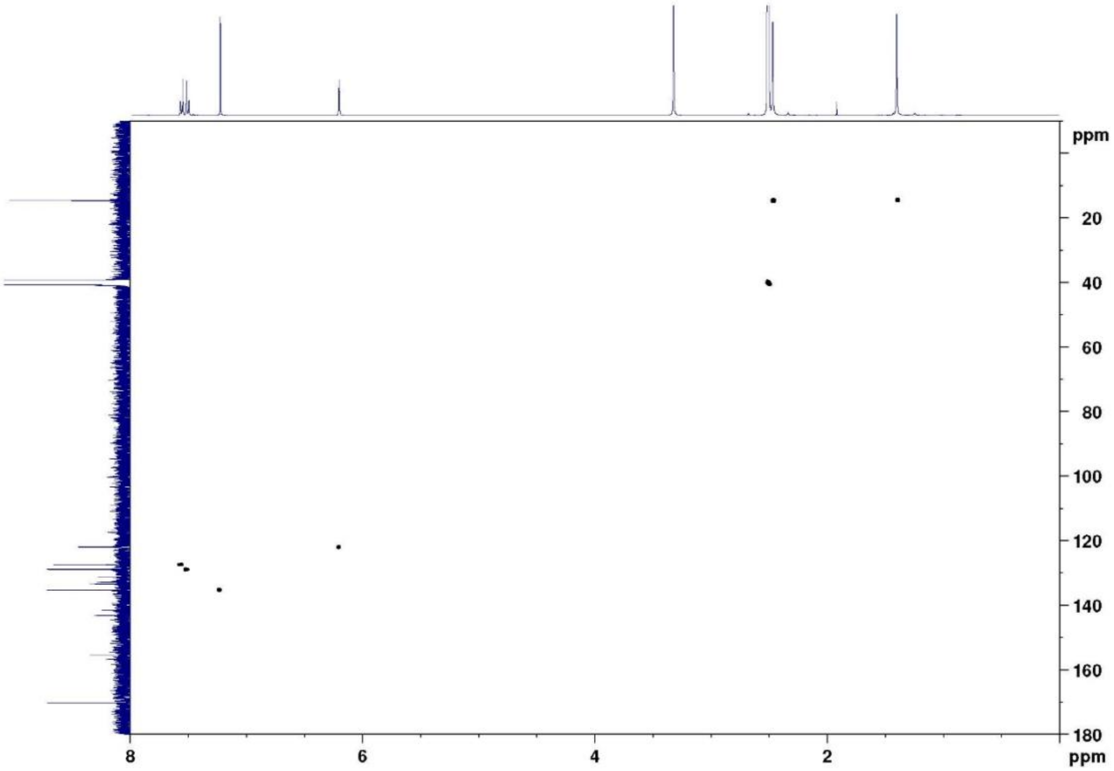

Supplement: Supplementary file 1 [file molecules-27-02455-s001.zip › molecules-1608555-supplementary.pdf]
